# Cooperation of Tyrosine Kinase Receptor TrkB and Epidermal Growth Factor Receptor Signaling Enhances Migration and Dispersal of Lung Tumor Cells

**DOI:** 10.1371/journal.pone.0100944

**Published:** 2014-06-24

**Authors:** Rudolf Götz, Michael Sendtner

**Affiliations:** Institute for Clinical Neurobiology, University Hospital Würzburg, Würzburg, Germany; Aix-Marseille University, France

## Abstract

TrkB mediates the effects of brain-derived neurotrophic factor (BDNF) in neuronal and nonnneuronal cells. Based on recent reports that TrkB can also be transactivated through epidermal growth-factor receptor (EGFR) signaling and thus regulates migration of early neurons, we investigated the role of TrkB in migration of lung tumor cells. Early metastasis remains a major challenge in the clinical management of non-small cell lung cancer (NSCLC). TrkB receptor signaling is associated with metastasis and poor patient prognosis in NSCLC. Expression of this receptor in A549 cells and in another adenocarcinoma cell line, NCI-H441, promoted enhanced migratory capacity in wound healing assays in the presence of the TrkB ligand BDNF. Furthermore, TrkB expression in A549 cells potentiated the stimulatory effect of EGF in wound healing and in Boyden chamber migration experiments. Consistent with a potential loss of cell polarity upon TrkB expression, cell dispersal and de-clustering was induced in A549 cells independently of exogeneous BDNF. Morphological transformation involved extensive cytoskeletal changes, reduced E-cadherin expression and suppression of E-cadherin expression on the cell surface in TrkB expressing tumor cells. This function depended on MEK and Akt kinase activity but was independent of Src. These data indicate that TrkB expression in lung adenoma cells is an early step in tumor cell dissemination, and thus could represent a target for therapy development.

## Introduction

Lung cancer is the second most commonly diagnosed cancer and the leading cause of cancer-related death among the malignant tumors [1]. More than a million deaths per year are due to lung cancer worldwide. Based on clinical pathology, 15–20% of lung carcinomas are categorized as small-cell lung cancer (SCLC) and 80–85% as non-small-cell lung cancer (NSCLC). NSCLCs are further divided into three different histological subtypes [2], adenocarcinoma (30–40%), squamous cell carcinoma (20-25%) and large-cell carcinoma (15–20%). NSCLC is initiated in lung cells by toxicity (e.g. from tobacco smoke) that causes genetic alterations. Additional molecular changes in premalignant cells result in advanced cancer and metastasis [3], [4]. The primary reason for the low cure rate from NSCLC is that about 70% of patients present with advanced disease, after the formation of metastatic spreading, and that even early stage NSCLC have a low overall survival rate [5]. Whether the metastatic cells disseminated from an aggressive NSCLC primary tumor at around the time of advanced stage disease or by clonal outgrowth of dormant micrometastatic cells that had dislodged from an early primary tumor years before first disease symptoms is an unresolved question [6]. In cases of advanced disease (spread to contralateral and mediastinal lymph nodes or even distant metastases) systemic chemotherapy is the main treatment.

Lung cancer progression depends on the capacity to invade and to metastasize to distant sites. Tumor cell metastasis is thought to be controlled by molecular processes that are different from those which control tumor initiation and growth [7]. Support for this hypothesis comes from the observation of human cancer lesions as well as several mouse models in which tissue-specific oncogene expression led to tumor initiation, yet tumor progression was not observed [8], [9]. The metastatic process is complex because it involves several distinct steps such as tumor cell dispersal from the epithelium, invasiveness, intravasation into lymph or blood vessels, dissemination, and extravasation into a remote organ and colonization of this organ [10].

Tropomyosin-related kinase TrkB (*Ntrk2*) and its ligand BDNF play an instrumental role in nervous system development [11]. BDNF/TrkB signaling stimulates migration of cerebellar granule cells and cortical neurons during development, and the absence of TrkB signaling during embryonic cortical development causes a delay in neuronal migration and thereby disturbes neuronal stratification [12]. Acquired TrkB (over)expression has been detected in various human cancers [13], including neuroblastoma [14], pancreas [15], colon [16], stomach [17] and multiple myeloma [18]. In a study on the expression of TrkB in NSCLC [19] about two thirds of the patients showed elevated TrkB expression. Notably, acquired expression of TrkB has been associated with metastasis and poor patient prognosis in NSCLC [19], [20] as well as in pancreas [15], gastric [17] and colon cancer [16]. Non-synonymous point mutations in the kinase and extracellular domains of TrkB have been found in several cancers, including colon cancer [21] lung adenocarcinoma [22] and large cell neuroendocrine carcinoma of the lung [23]. Because these mutations were identified by large scale sequencing approaches of cancer samples, the variant proteins could be either passenger or driver mutations and the discrimination between these two possibilities requires subsequent analysis of the transforming activity of the protein variants. This evaluation has been done recently for two of the TrkB mutations found in colon cancer, one mutation found in a lung adenocarcinoma cell line [24] and three mutations detected in large cell neuroendocrine carcinoma of the lung [25]. The mechanism how these TrkB variants could contribute to tumorgenicity remained unclear from these studies.

Previous studies to dissect the functional effects have shown that forced expression of TrkB in rat intestinal epithelial cells conferred anoikis resistance *in vitro* and allowed the TrkB expressing cells to form tumors and metastases in nude mice [26], [27]. Further experiments using TrkB/BDNF expressing rat intestinal epithelial cells demonstrated that TrkB/BDNF induced epithelial-mesenchymal transition (EMT) through regulation of E-cadherin expression that required the transcription factor Zeb-1 in order to suppress E-cadherin expression [28].

In this study we examined the effects of TrkB expression in two human lung adenocarcinoma cell lines on fundamental properties of metastatic cells, including cell migration, cell spreading and invasiveness. We found that TrkB activation enhanced migration and dispersal of these cells. TrkB was not only activated by BDNF, but also could be transactivated by EGF receptor (EGFR) signaling, as recently shown in early neurons that form the cortical layers of the brain [29]. These data indicate that TrkB could play a central role in early steps of metastasis.

## Results

### Expression of TrkB in lung tumor cells enhances cell migration and wound closure

**Figure 1:**
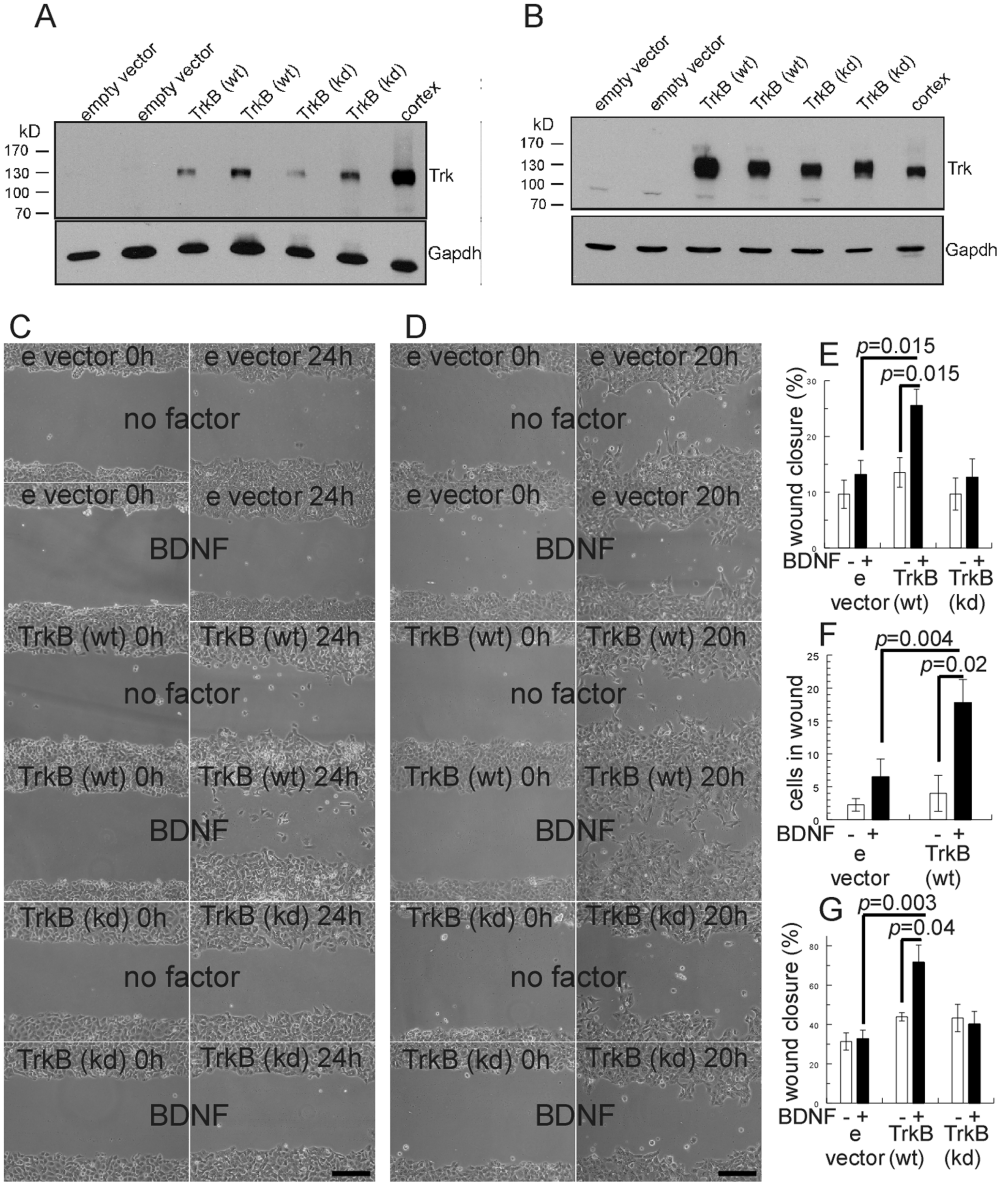
Expression of TrkB in human adenocarcinoma cells enhances cell motility in wound closure. A, B. Immunoblot analysis of cell lysates from A549 cell clones (A) or NCI-H441 cell clones (B) transduced with retrovirus harbouring empty vector or vector encoding TrkB and kinase-dead TrkB (kd), respectively. TrkB expression was evaluated with a COOH-terminal (C14) pan-Trk antibody. Adult mouse brain cortex tissue served as positive control. Gapdh served as a loading control. C, D. Wound closure assay demonstrating increased cell motility of A549 cells (C) or NCI-H441 cells (D) expressing TrkB. Cells were plated in 6-cm dishes and grown to 100% confluence. Plates with serum-starved confluent cells were scratched with a pipette tip to create a single scratch wound, washed and cultured for another 24 h (A549 cells) or 20 h (NCI-H441 cells) in the absence or presence of BDNF (10 ng/ml). Representative photographs were taken immediately after scratch injury and 20 h or 24 h later. Bar, 200 µm. E. Bar graphs show quantification of wound closure of A549 cell clones. F. Bar graphs show number of live cells of A549 cell clones in the wound area. G. Bar graphs show quantification of wound closure of NCI-H441 cell clones. Bars, mean ± SEM from three independent cell clones.

The overexpression of tropomyosin receptor kinase B has been observed in several aggressive cancers, including NSCLC [13], [19], [30]. Of note, TrkB expression correlated significantly with metastasis and poor prognosis in NSCLC [19], [20]. Furthermore, TrkB was identified in an unbiased screen for genes that suppress programmed cell death when epithelial cells were deprived from attachment to extracellular matrix [26]. To examine whether and how TrkB expression could support tumor cell dissemination and metastatic progression in NSCLC, we used two distinct NSCLC cell lines, A549 and NCI-H441. To mimic the *in vivo* situation, we overexpressed TrkB in these cell lines. We cloned wild-type and, as a negative control, kinase-inactive mutant TrkB (unable to bind ATP [31]) into the retroviral pBABE-puro expression vector. We transduced virus harbouring these TrkB vectors or empty vector control virus independently into both lung cancer cell lines and isolated under puromycin selection several independent individual clones from each transduction. We confirmed by western blot analysis that TrkB and kinase-dead TrkB proteins, respectively were expressed in several independent A549 (Figure 1A) and NCI-H441 (Figure 1B) clones at levels comparable to those observed in mouse brain tissue. Empty vector clones did not reveal a Trk signal indicating absence of endogenous TrkB expression. We thus generated a cell system allowing us to evaluate the activities and functions of TrkB expression in lung cancer cells.

In order to study the effects of TrkB expression on cell migration, a scratch wound assay was used. Incubation of A549 empty vector or A549 cells expressing a kinase-dead TrkB showed a modest effect on wound repair (around 10–15% wound closure within 24 hours, Figure 1C and E) which was not significantly increased by the addition of BDNF. This lack of response in clones harbouring empty vector is expected because there was no Trk expression detectable by Western blot analysis (Figure 1A). In contrast, incubation of TrkB expressing cells with BDNF stimulated lung cancer cell migration in the wound closure assay (Figure 1C and E). Besides augmented wound closure, BDNF addition to TrkB expressing cells but not to cells expressing kinase-inactive TrkB, induced single cells or small cell clusters in the wound area (Figure 1C and F). A significant stimulatory effect of BDNF on wound closure was also observed in NCI-H441 clones where the expression of TrkB enhanced BDNF-dependent cell migration whereas clones carrying empty vector or expressing kinase-dead TrkB showed a lower level of wound closure (Figure 1D and G).

### TrkB expression acts synergistically with EGF to stimulate migration

**Figure 2:**
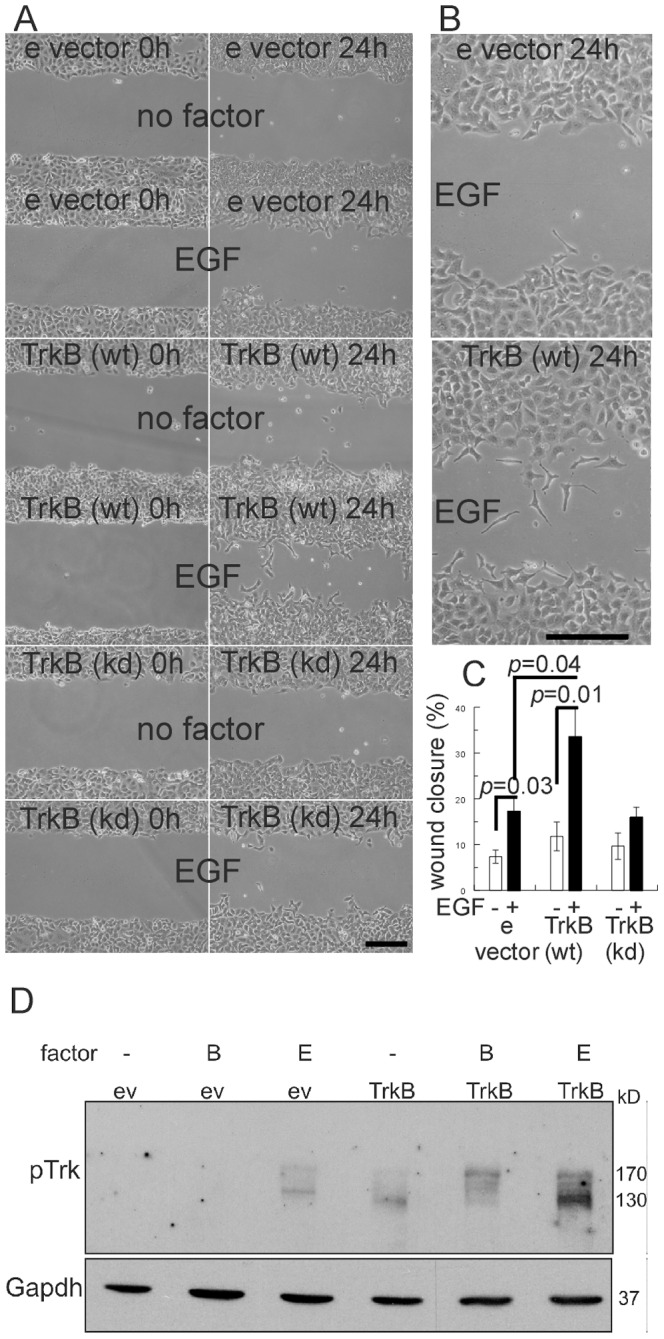
TrkB expression acts synergistically with EGF to stimulate migration. A. Wound closure assay demonstrating increased cell motility of A549 cells expressing TrkB upon EGF stimulation. Cells were plated in 6-cm dishes and grown to 100% confluence. After generating a single scratch wound in the monolayer, cells were washed and cultured for another 24 h, Representative photographs were taken immediately after scratch injury and 24 h later. Bar, 200 µm. Bar graphs show quantification of wound closure and number of live cells in the wound area. Bars, mean ± SEM (n = 6). Bar, 200 µm B. Higher magnification picture demonstrating the effect of TrkB expression in response to EGF, note spindle-shaped morphology of cells in the wound area in response to EGF. Bar, 200 µm C. Bar graphs show quantification of wound closure in response ot EGF. Bars, mean ± SEM (n = 6). D. Phosphorylation of TrkB in response to BDNF and EGF. Immunoblot analysis of equal amounts of cell lysates from A549 empty vector (ev) and TrkB cell clones using a phospho-specific antibody that recognizes phosphorylated tyrosines within the catalytic domain of TrkB (pY674/pY675 in human TrkA). Gapdh served as a loading control.

Next, we tested the effect of EGF in the wound closure response. We observed that TrkB expression significantly enhanced the effect of EGF in wound repair, as compared to the stimulatory effect of EGF in empty vector and TrkB (kinase-dead) cell clones (Figure 2A and C). A significantly increased number of single cells with spindle-like morphology was found in the wound area of TrkB expressing cells treated with EGF (Figure 2B). TrkB activation was analysed in extracts of A549 cells plated in subconfluent conditions and treated with BDNF or EGF for 5 minutes. In response to BDNF, bands of 130 and 170 kD were visible, as reported previously for neural cells [29]; these bands were absent in empty vector transfected cells (Figure 2D). In the absence of BDNF, the 170 KD band was weaker, while the 130 kD band was more intense. Cell extracts from TrkB expressing cells treated with EGF also showed multiple bands, including those of 130 and 170 kD; EGF addition to cells harbouring empty vector also revealed a 140 kD band that was absent in empty vector cell extracts not treated with growth factor or treated with BDNF (Figure 2D).

**Figure 3:**
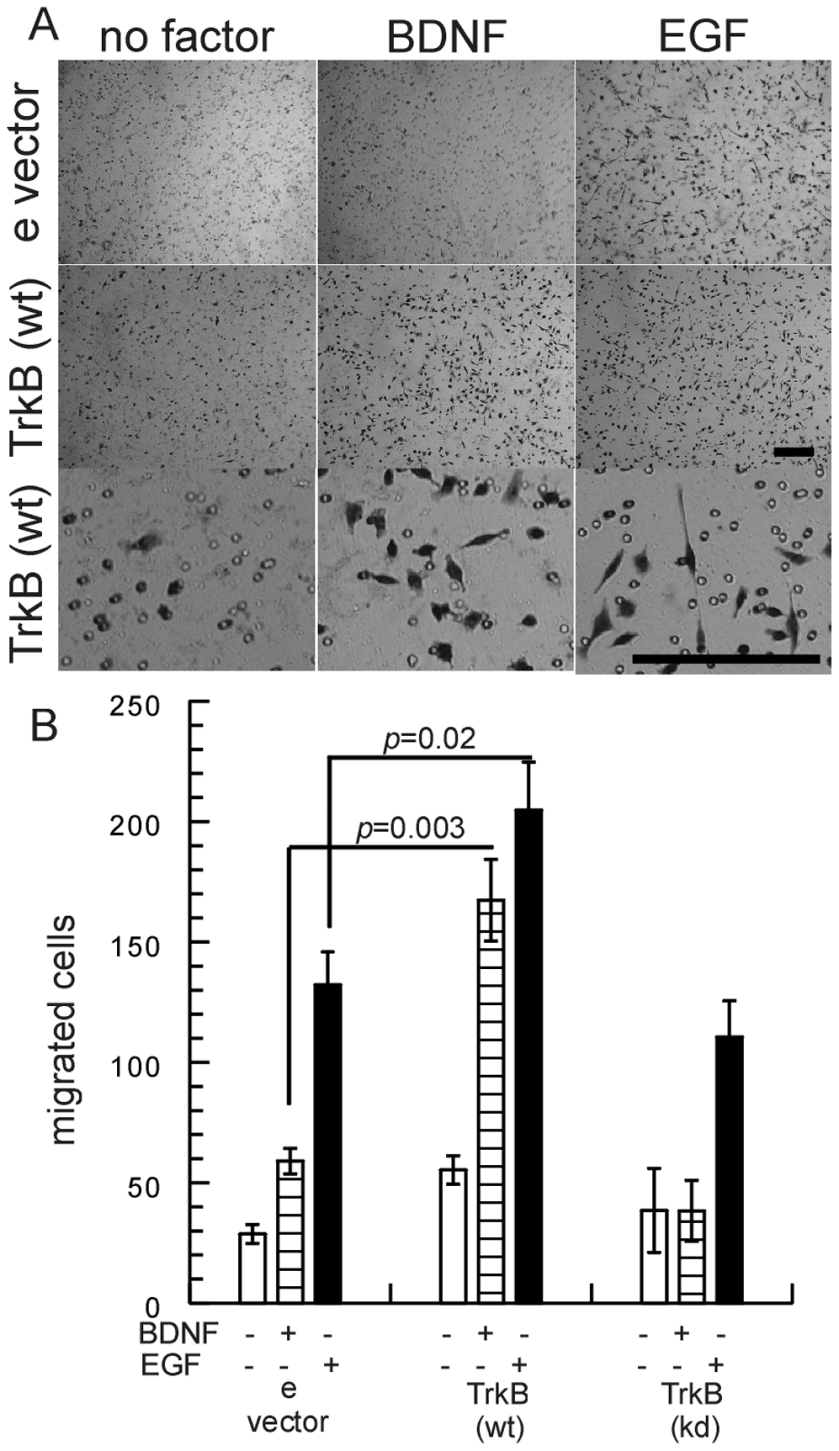
TrkB expression confers increased migration in transwell assays. A549 cells (2×10^4^) suspended in culture media were seeded in the upper chamber of transwell plates. After 18 h incubation, cells that migrated to the lower surface of the filters were fixed, stained with cresyl violet and quantiified by counting three randomly selected fields using a 10x objective. Bar, 200 µm. Bars, mean ± SEM.

To support the above findings from the wound closure assay, we evaluated the migratory capacity of TrkB expressing cells in Boyden chamber assays. As shown in Figure 3, A549 cells expressing wild-type TrkB invaded through the collagen-coated filters upon BDNF stimulation. It is of note, that the stimulatory effect of EGF in transwell migration of A549 empty vector and Trk-kinase-dead expressing cells was significantly enhanced by the expression of TrkB (Figure 3). Thus, TrkB expression in A549 cells did not only mediate increased migration upon stimulation with BDNF, but in addition potentiated the action of EGF in wound repair and transwell migration experiments.

### TrkB expression mediates cell spreading

**Figure 4:**
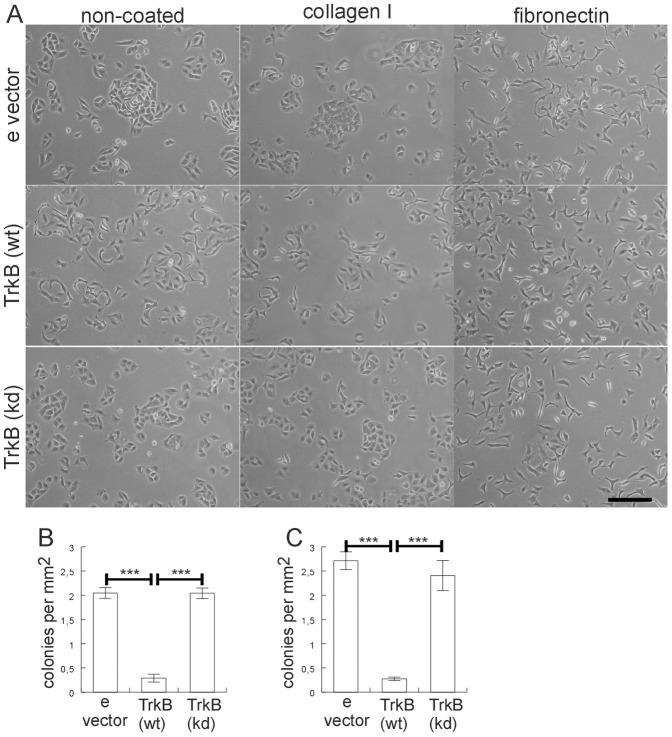
TrkB expression induces cell spreading dispersal. A. Morphology of A549 cell colonies and cell dispersal harbouring empty vector (e vector), or expressing wild-type TrkB (wt) or kinase-dead TrkB (kd) plated on noncoated, collagen I or fibronectin-coated dishes. Phase contrast pictures were taken using a 10x objective. Bar, 200 µm B. Bar graphs show quantification of colonies on non-coated dishes. Bars, mean ± SEM (n = 6). C. Bar graphs show quantification of colonies on collagen I-coated dishes. Bars, mean ± SEM (n = 6); (***, p<0.0002).

In order to further investigate the potential loss of cell polarity upon TrkB expression (see Figure 2B), we plated lung tiúmor cells at moderate density (15.000 cells per cm^2^) on serum-coated plastic or collagen I-coated surfaces. A549 cells formed compact colonies 24 hours after plating with a clustered cobblestone-like appearance, typical for many epithelial cells cultured in the absence of pro-inflammatory cytokines. This allowed us to study the effect of TrkB on de-clustering and cell dispersal. A549 cell clones expressing TrkB grown on a serum-coated cell culture-plastic surface showed a dispersal and scattering of the compact colonies with apparently reduced cell-cell contacts (Figure 4A and B). This morphologic transformation was observed in the absence of BDNF addition in independent clones expressing TrkB, and was neither observed in the empty vector clones nor in the clones expressing kinase-inactive TrkB (Figure 4A and B). In order to exclude the possibility that unwanted alterations that inhibited declustering had occurred in any of the cell clones during selection or passaging, cells were also grown on fibronectin which leads to cell dispersal and EMT in primary alveolar epithelial cells as reported previously [32]. Culturing the cell clones on fibronectin revealed a similar extent of cell dispersal in all clones arguing against passaging-induced alterations (Figure 4A). A549 cell clones expressing TrkB grown on a collagen I coated surface also showed a dispersal of the compact colonies with apparently reduced cell-cell contacts (Figure 4A and C). Notably, many individual TrkB expressing cells grown on plastic/serum or collagen (Figure 4A) showed a more elongated and sometimes even a spindle-shaped morphology. The morphological transformation appeared as robust as in human breast epithelial fibrocystic MCF10A cells, but less pronounced than that observed in RIE-1 rat intestinal epithelial and RK3 rat epithelial cells, where TrkB/BDNF expression induced a strong EMT [33].

**Figure 5:**
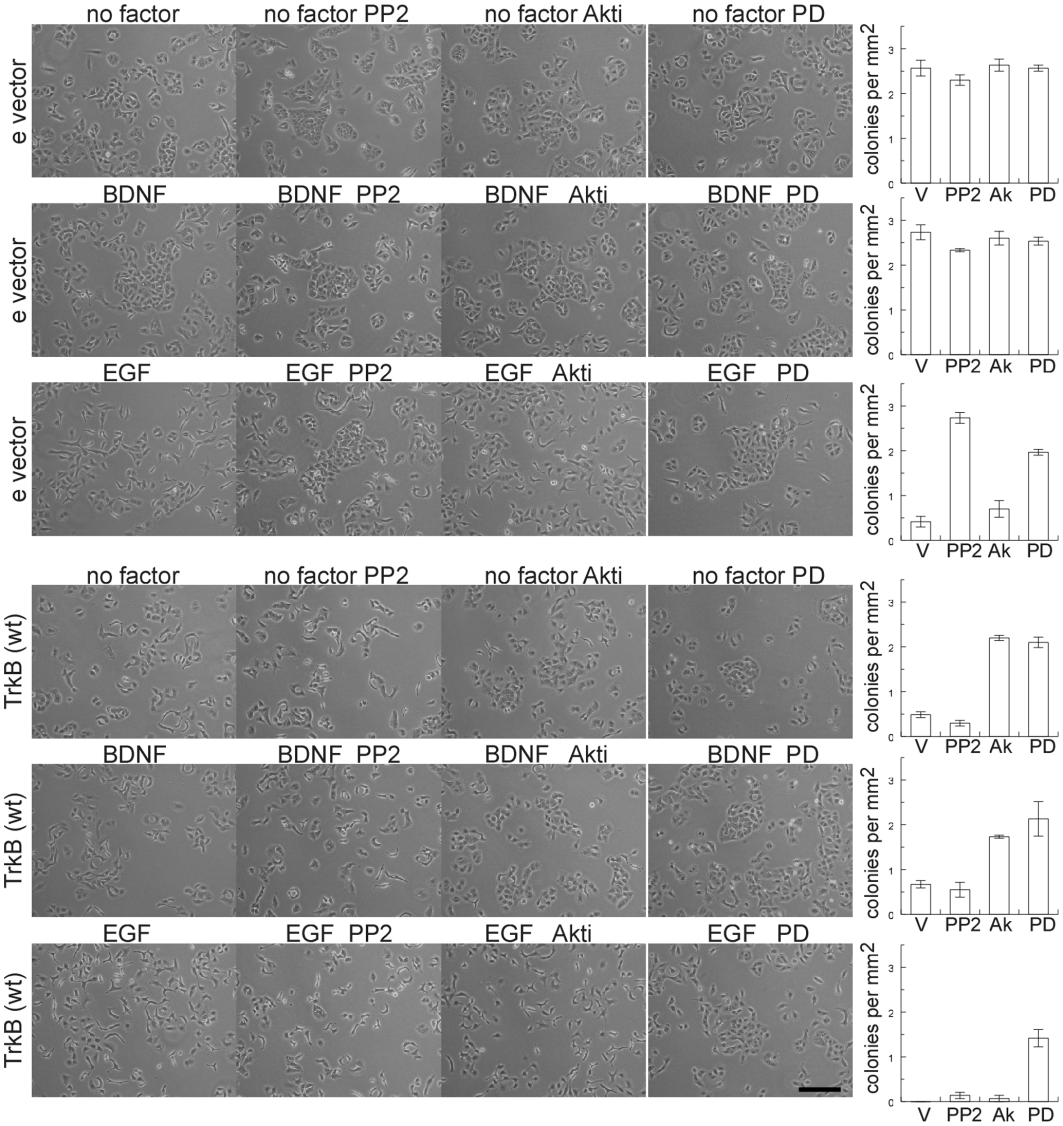
TrkB expression induced cell spreading requires Akt and MEK kinase activity. Morphology of cell colonies and cell dispersal of A549 cells harbouring empty vector (e vector) or expressing wild-type TrkB (wt). Cells were plated on noncoated dishes, grown for 24 hours in the presence of 0.5% serum and subsequently stimulated with BDNF or EGF for a period of 24 hours in the presence or absence of Src inhibitor PP2, Akt kinase inhibitor Akti-1,2 (Akti) or MEK inhibitor PD0325901 (PD); untreated cells were cultured in the presence of vehicle (v, 0.1% DMSO). Bar, 200 µm. Bar graphs show quantification of colonies. Bars, mean ± SEM (n = 3).

To elucidate the signaling pathways involved in cell spreading of A549 cell clones expressing TrkB, cells were cultured in the presence of inhibitors. Blockage of the Raf/MEK/ERK pathway, or the Akt and Src kinases was achieved by the addition of 1 µM PD0325901, 1 µM Akti-1,2, and 1 µM PP2, respectively. Empty vector clones formed compact colonies without growth factor addition and in the presence of BDNF, irrespective of the presence of inhibitors (Figure 5). The addition of Akti-1,2 and PD0325901 suppressed the dispersal phenotype in TrkB expressing cells, and the addition of BDNF did not alter the compact phenotype (Figure 5), demonstrating that TrkB-induced cell dispersal is reversible within 24 hours of growth and depends on Akt and MEK kinase signaling pathways. The Src kinase inhibitor PP2 was unable to ablate the dispersed morphology in TrkB expressing cells.

Next, we examined the effect of EGF on cell spreading in TrkB and empty vector cells. As expected, stimulation with 10 ng/ml EGF for 24 hours induced cell spreading (Figure 5). In cells harbouring empty vector, addition of PP2 and PD0325901 suppressed the EGF-induced morphological changes and the cells displayed a clustered phenotype, whereas Akti-1,2 had no significant influence on EGF-induced cell dispersal (Figure 5). The de-clustering of TrkB expressing cells exposed to EGF was unaffected by PP2 and Akti-1,2 but inhibited by PD0325901 (Figure 5). Thus, while TrkB requires Akt and MEK kinase to induce de-clustering, the activation of EGF in combination with TrkB expression relieves cells from dependence on Src and Akt. In other words, whereas Src and MEK kinases were instrumental for spreading of A549 cells in response to EGF, Src was no longer required when TrkB was co-expressed.

### TrkB expression causes disruption of membrane-associated E-cadherin

**Figure 6:**
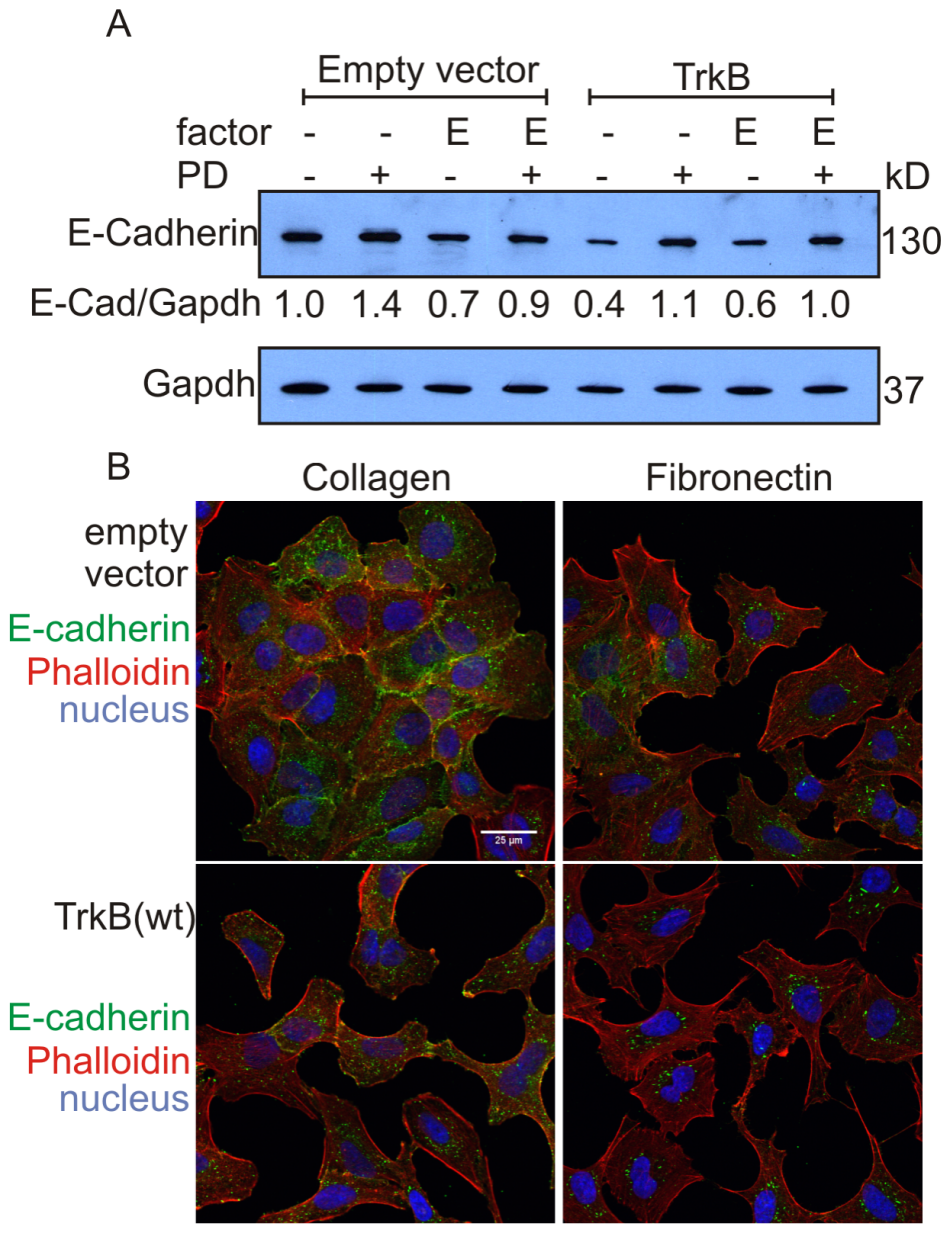
TrkB expression suppresses cell-surface expression of E-Cadherin. A. Immunoblot analysis of cell lysates from A549 cell clones stably expressing TrkB or harbouring empty vector, cultured on plastic in the absence or in the presence of EGF (E), and with or without the MEK inhibitor PD0325901 (PD), respectively. Expression levels of E-cadherin were quantified by densitometric analysis and normalized by comparison to the Gapdh loading control, shown for each lane as values. B. Immunostaining of E-cadherin and F-actin. Cells were grown on collagen I or fibronectin coated glass slides, serum deprived for 24 hours, fixed and stained. Nuclei were counterstained with DAPI. Results are representative of more than three experiments.

Since expression of TrkB in A549 cells suppressed the formation of clustered cells and thus deregulated their polarity, we asked whether the strength of cell-cell contacts is also reduced after elevated TrkB expression. Downregulation of E-cadherin expression is characteristic of early epithelial to mesenchymal transition [34]. Thus, we investigated whether the level of E-cadherin in TrkB expressing A549 clones was altered compared to control clones harbouring empty vector. We determined the levels of cadherin expression by western blotting of cell lysates prepared from cells that showed dispersal, or no dispersal (TrkB expressing cells treated with the MEK inhibitor PD0325901). Notably, E-cadherin protein levels were reduced approximately twofold in TrkB expressing cells, compared to cell clones harbouring empty vector (Figure 6A). Treatment with PD0325901 restored the expression of E-cadherin, in line with a switch from cell dispersal to a compact cell morphology (see Figure 5). A similar level of reduced E-cadherin expression was observed upon culture of TrkB expressing cells in the presence of EGF.

In order to further delineate the mechanism of cell dispersal, we investigated cytoskeletal changes and determined the subcellular localization of E-cadherin. In control clones, E-cadherin was localized along the intercellular surfaces, indicative of the existence of E-cadherin-mediated interactions among neighbouring cells. TrkB expressing clones showed a reduction of E-cadherin staining at cell-cell contacts and an increased punctuate staining throughout the cytoplasm suggesting that E-cadherin is either endocytosed or not delivered to sites of cell-cell contacts (Figure 6B). Cells cultured on a fibronectin substrate, which induced cell spreading (see Figure 4A, right column) showed a similar pattern of internal E-cadherin expression. The arrangement of the actin filaments, visualized by phalloidin staining, revealed that TrkB expression promoted extensive cytoskeletal changes (Figure 6B). A549 cells carrying empty vector exhibited highly organized filamentous actin immediately underneath the cell membrane. TrkB expression resulted in a redistribution of F-actin with evidence of F-actin stress fibre formation (Figure 6B).

## Discussion

We show here that forced expression of the TrkB receptor kinase in two human lung adenocarcinoma cell lines, A549 and NCI-H441, caused enhanced migratory capacity in the presence of the TrkB ligand BDNF. Furthermore, TrkB expression in A549 cells potentiated the stimulatory effect of EGF in wound healing and in Boyden chamber migration experiments. TrkB expression conferred cell dispersal and de-clustering of A549 cell colonies that did not require the addition of exogeneous BDNF and depended on MEK and Akt kinase function but was independent of Src signaling. This morphological transformation was found to correlate with a reduction of E-cadherin expression and a suppression of E-cadherin expression at the cell surface in cells expressing TrkB. From these data we envision an *in vivo* scenario, where lung adenoma cells would be able to dislodge from the epithelium and become invasive once TrkB becomes expressed.

The failure of NSCLC therapy is mostly due to the development of local and distant metastasis [35]. Dissemination of cells from the primary tumor is considered a major step and requires loss of cell contact to neighbour cells as a first step to escape the epithelium of origin. A549 and NCI-H441 cells are a well established *in vitro* cell system valuable to explore the transitions that occur during NSCLC progression. Our observation that TrkB expression in A549 cells induced cell dispersal and prevented the formation of compact cell clusters in the absence of exogenous BDNF is novel. The finding that TrkB expressing tumor cells showing cell dispersal retained E-cadherin intracellularly is compatible with observations from clinical carcinoma specimens [36]. Alternatively, E-cadherin can be suppressed at the transcriptional level [33]. Maintenance of E-cadherin expression might actually provide an opportunity for the migrating tumor cell to attach again in a new location and form a micrometastasis when a suitable environment is encountered. In A549 cells it has been shown, that FGF-1 could restore E-cadherin expression and revert EMT induced by TGF-β1 [37]. During the invasion and migration phases, TrkB provides protection from anoikis [26]. The mechanism behind the cell dispersal phenotype is unclear. It might involve activation of TrkB by the EGFR, as shown for neural precursor cells [29]. Alternatively, transactivation of the EGFR is a well-known mechanism that can occur either through non-receptor tyrosine kinases or through EGFR ligand shedding mediated by metalloproteases [38].

The extent of cell dispersal induced by BDNF in epithelial cells with ectopic TrkB expression was found to be variable in different cell lines derived from intestine, kidney and breast [33]. Furthermore, different conditions of cell culture such as cell density may also affect the extent of cell dispersal. In contrast to cell dispersal, cell migration required the addition of exogenous BDNF to TrkB expressing lung tumor cells. The source of BDNF may be the tumor microenvironment [39]. BDNF produced by platelets [40] and/or endothelial cells [41] would be able to stimulate the migratory capacity and survival of the detached tumor cells.

A previous study noted the expression of TrkB in A549 cells [19] but we were unable to detect endogenous Trk expression by immunoblotting. Furthermore, the addition of BDNF did not augment wound closure in A549 control cells harbouring empty vector, indicating an absence of a positive effect of BDNF in wound closure. As reported previously, A549 cells respond to EGF by endocytosis of E-cadherin [42] and to TGF-β, a well-studied inducer of EMT, by suppression of E-cadherin expression [43], [44]. In our study, the expression of TrkB had a comparable molecular effect on E-cadherin in the absence of forced ligand expression or ligand addition.

Trk family members and their neurotrophin ligands regulate neuronal survival, differentiation and migration as well as synaptic plasticity [11]. In neuroblastoma, TrkB/BDNF expression is preferentially found in aggressive tumors, whereas the expression TrkA or TrkC is associated with better prognosis [45]. An explanation for this different cellular response may be that TrkA and TrkC, in contrast to TrkB, are dependence receptors that instruct developing neurons to die in the absence of ligand [46]. TrkA expression indeed induced cell death of neuroblastoma cells and required CCM2 as a primary effector [47].

Acquired TrkB (over)expression has been detected also in various human epithelial cancers [13]. Higher expression levels of TrkB have been found to be associated with aggressive tumor behaviour and poor prognosis in several epithelial cancers, including colon cancer [16], pancreatic cancer [15] and gastric cancer [17]. In NSCLC, TrkB expression correlated with lymph node metastasis [19], vascular invasion and poor disease-free and overall survival [20].

Taken together, these data indicate that TrkB could be a regulator of cell dispersal and migration and initiation of metastasis in many types of epithelial tumors, thus resembling a physiological function of this receptor in the developing brain. TrkB is expressed in early neurons before they migrate out of the subventricular zone in the cortical neuroepithelium towards the mantle zone where these neurons then end their migration in the cortical layers. Interestingly, this migration is dependent on TrkB and the activation of this receptor [12], [48], but does not depend on the presence of BDNF, indicating that nonligand mediated activation mechanisms are involved. Only later in development after neuronal migration has been finished these cortical neurons are activated by BDNF. This seems to be the case also for TrkB expressing A549 cells which start to detach from the epithelial structure by morphological transformation in a BDNF independent manner, and subsequently respond to BDNF in their migration. The initial start of migration depends on TrkB activation, and it is highly suggestive that other signaling pathways described previously to transactivate TrkB are involved, as previously shown for early cortical neurons that start to migrate [29]. These results indicate that interference with TrkB expression and activation could be a means to inhibit dispersal and migration of these tumor cells and thus to prevent initiation of migration. This could be a potential new approach for NSCLC therapy.

## Materials and Methods

### Cell culture

The human NSCLC cell line A549 (bronchioalveolar carcinoma, American Type Culture Collection, ATCC-CCL-185, [49]) was cultured in Dulbecco's Modified Eagle Medium (DMEM) supplemented with 10% fetal bovine serum and Pen/Strep; a second human lung adenocarcinoma cell line NCI-H441 (ATCC-HTB-174, [50]) was propagated in RPMI 1640 medium with 10% FCS and Pen/Strep. Cells were cultured at 37°C, 5% CO_2_. Both cell lines were subjected to DNA typing of short tandem repeats and genotyped to ensure their identity (Leibniz-Institute DSMZ). DMEM, RPMI-1640, FCS, Glutamax and Pen/Strep were purchased from Life Technologies. All cells were cultured on tissue culture-treated plastic and rat tail collagen I (50 µg/ml, BD Biosciences) or fibronectin-coated (50 µg/ml, R&D Systems) glass, respectively at 37°C and 5% CO_2_ atmosphere. For migration assay, cells were made quiescent by culture in fresh medium containing 0.5% serum for 1 day.

### Generation of stable cell lines, plasmids and primers

Retrovirus was produced in HEK293T cells by transfection. The pBABE-puro vector (Addgene #1764) contained the full length murine TrkB cDNA. A point mutant (K571M, corresponding to K560M in rat TrkB) encoding a kinase-inactive TrkB [31] was constructed using a commercial kit (Agilent quickchange II site-directed mutagenesis kit) according to the manufacturer's protocol. Oligonucleotides were as follows, sense, GATCCTGGTGGCTGTGATGACGCTGAAGGACGCC, and antisense, GGCGTCCTTCAGCGTCATCACAGCCACCAGGATC. The mutation was confirmed by sequencing.

Two million cells grown on a 10-cm dish were transfected using Lipofectamin 2000 (Invitrogen) with 9 µg vector DNA, 8 µg pUMV3a expression plasmid encoding gag/pol (Addgene #8449) and 1 µg CMV-VSV-G plasmid (Addgene #8454). Cell culture supernatant containing the retrovirus was harvested 60 h after transfection, mixed with polybrene (10 µg/ml; Santa Cruz Biotechnology), filtered through 0.45 µm filters and used for transduction of A549 and NCI-H441 cells. Three days after transduction, different aliquots of the cells were seeded and puromycin (Santa Cruz Biotechnology) selection was begun (2.5 µg/ml for A549 cells, and 1 µg/ml for NCI-H441 cells). Individual colonies were isolated using cloning cylinders (Millipore).

### Protein extraction and immunobloting

For Western blot analysis, cells were washed twice with phosphate-buffered saline and protein was extracted with a lysis buffer consisting of 50 mM Tris-HCl (pH 7.5), 150 mM NaCl, 2 mM EDTA, 1% (v/v) Nonidet P-40, 0.5% (w/v) sodium deoxycholate in the presence of proteinase inhibitors (cOmplete Protease Inhibitor Cocktail, Roche) and a phosphatase inhibitor cocktail (PhosSTOP, Roche). Cell extracts were clarified by centrifugation at 20,000×g, and the supernatant was subjected to protein content determination using a BCA protein assay kit (Pierce). SDS/polyacrylamide gel electrophoresis and electroblotting to nitrocellulose membranes (Whatman Protran BA83, 0,2 µm) were done as described [51]. The following antibodies were used: rabbit anti-Trk (Santa Cruz Biotechnology, sc-11, 1∶1.000); rabbit anti-phospho-TrkA (pTyr674/675) (Cell Signaling, 4621, 1∶1.000); rabbit anti E-cadherin (Cell Signaling 3195, 1∶2.000); 1∶2.000, mouse anti GAPDH (Calbiochem, CB1001, 1∶3.000). The membranes were developed by chemiluminescence detection using ECL or ECL prime (GE Healthcare) with a goat horseradish peroxidase-conjugated secondary antibody. The images were recorded on x-ray film and then scanned on a flatbed scanner. Band intensities were quantified using Image J.

### Cell aggregation and migration assays

For cell aggregation, cells were harvested from 80% confluent cultures with trypsin and plated at a density of 15.000 cells/cm^2^ on 9-cm dishes (Nunc). After 24 h, the cultures were fed with new medium containing 0.5% foetal calf serum and further cultured for 24 h. Then, inhibitors were added 1 h prior to stimulation with growth factors, as indicated, and remained in the medium for the remainder of the experiment. Akti-1,2, PD0325901 and PP2 were purchased from Calbiochem and used at 1 µM. BDNF or human EGF (Peprotech; 10 ng/ml) were added for evaluation of cell-declustering and the culture was incubated for 24 h. Photographs were taken (Nikon Eclipse TS100 inverted microscope equipped with a 10x 0.24A phase contrast lens and a DS-2Mv camera). The number of cell clusters with a diameter larger than 125 µm was quantified; values are representative of at least 40 microscopic fields (area 0.7946 mm^2^ each) from three independent experiments.

For wound healing, cells were seeded at a density of 1.5 Mio in 6-cm plates (BD Biosciences) until a confluent monolayer had developed. At confluency, medium containing 0.5% foetal calf serum was added to the cultures and incubation continued for 24 h, before being wounded with the use of a 200 µl pipette tip that extended the full diameter of the dish. Detached cells were removed from the cultures by two rounds of washing with HBSS, followed by incubation with medium containing 0.5% foetal calf serum supplemented with or without BDNF or EGF (10 ng/ml). Images of the wound were photographed with a 10x lens immediately following wounding and 20 h (for NCI-H441 cells) or 24 h (for A549 cells) later. The number of phase-bright living cells present in the wound was counted and the wound area in each image was determined. Wound closure was quantified using ImageJ by following the change in wound area over time. Values are representative of between five and nine experiments.

Boyden chamber cell migration was carried out in transwell plates (Corning, 24-well, 8 µm pore size). The upper surface of the filter was precoated with collagen I (50 µg/ml). A549 cells were grown to 70% confluency, followed by a 24 h culture in medium containing 0.5% foetal calf serum. Cells were harvested with trypsin and placed in the upper chamber at 20.000 cells in 200 µl of medium containing 0.5% foetal calf serum. BDNF of EGF (10 ng/ml) were used as chemoattractant in the lower chamber filled with 600 µl medium containing 0.5% foetal calf serum, and the cells were allowed to migrate for 12 hours. Cells that had migrated onto the lower surface of the membrane were fixed with 4% (w/v) paraformaldehyde, stained with eosin, and quantified by microscopy. At least three separate microscopic fields were counted per filter.

### Immunofluorescence microscopy

Cells were grown on cover glasses (10 mm diameter) coated with rat tail collagen I (50 µg/ml) or fibronectin-coated (50 µg/ml) in 4-well dishes (Nunc) at a density of 15.000 cells/cm^2^ under conditions described above. Cells were fixed for 15 min with 4% (w/v) paraformaldehyde, washed with PBS. After a 90-min incubation with permeabilization buffer (PBS with 5% goat serum and 0.3% Triton X-100), primary antibodies (mouse anti E-Cadherin, BD Biosciences, 610181, 1∶200) were added for 120 min. The cells were washed four times with wash buffer (PBS with 0.2% Triton X-100 and 0.1% Tween 20) followed by incubation with Alexa Fluor-488-labelled antibody (Invitrogen, 1∶800). For F-actin staining, rhodamin-phalloidin (Cytoskeleton Inc.) was used at a concentration of 70 nM. DAPI (1 µg/ml) was used to counterstain cell nuclei. Images were obtained using a confocal laser scanning microscope (Olympus FluoView FV1000 confocal LSM) equipped with a UPLFLN 40xO NA: 1.30 lens. Images represent a single confocal section. Images were processed with Image J.

### Statistical analysis

One-way analysis of variance (ANOVA) was applied for the comparison of samples. Data are presented as mean ± standard error of the mean (SEM). Statistical significance was assessed by calculating the *P* values; *P*<0.05 was considered significant).
